# Modified American Joint Committee on Cancer Tumor-Node-Metastasis Staging System Based on the Node Ratio Can Further Improve the Capacity of Prognosis Assessment for Gastric Cancer Patients

**DOI:** 10.3389/fonc.2019.00329

**Published:** 2019-05-03

**Authors:** Ze-Long Yang, Ming-Hua Zhu, Xiu-Jing Han, Qiang-Wei Liu, Jian-Hai Long, Chun-Xi Wang

**Affiliations:** ^1^Department of General Surgery, Chinese People's Liberation Army General Hospital, Beijing, China; ^2^Department of General Surgery, Hainan Hospital of PLA General Hospital, Sanya, China; ^3^Clinical Laboratory, The First Affiliated Hospital of Guangzhou Medical University, Guangzhou, China; ^4^Anesthesiology and Operation Center, Chinese People's Liberation Army General Hospital, Beijing, China; ^5^Department of General Surgery, People's Liberation Army No. 520 Hospital, Mianyang, China

**Keywords:** gastric adenocarcinoma, tumor-node-metastasis, tumor-node ratio-metastasis, lymph nodes examined, survival

## Abstract

**Background and Objectives:** Our aim was to investigate whether the modified American Joint Committee on Cancer (AJCC) tumor-node-metastasis (TNM) staging system based on the node ratio can further improve the capacity of prognosis assessment for gastric cancer (GC) patients regardless of the number of lymph nodes examined (eLNs).

**Methods:** A total of 17,187 GC patients in the Surveillance, Epidemiology, and End Results (SEER) database were included. On the basis of a training set of 7,660 GC patients, we built the tumor-node ratio-metastasis (TNrM) staging system, which was then externally validated with a validation set of 9,527 GC patients.

**Results:** For the training set, the C-index value of the TNrM staging system was significantly higher than that of the AJCC 8th TNM staging system to predict survival for GC patients (C-index: 0.688 vs. 0.671, *P* < 0.001). Moreover, the C-index value of the TNrM staging system was significantly higher than that of the 8th TNM staging system to predict survival for GC patients with ≤15 eLNs (C-index: 0.682 vs. 0.673, *P* < 0.001), as well as for GC patients with >15 eLNs (C-index: 0.700 vs. 0.694, *P* < 0.001). Similar results were found in the validation set.

**Conclusions:** The TNrM staging system predicted survival more accurately and discriminatively than the AJCC 8th TNM staging system for GC patients regardless of the number of eLNs.

## Introduction

Gastric cancer (GC) is the second leading cause of cancer-related death and the fifth most common cancer worldwide (1). An accurate and discriminative staging system is necessary for doctors to assess the prognosis of patients and to make appropriate medical decisions. The most commonly used staging system for GC is the International Union Against Cancer (UICC) or American Joint Committee on Cancer (AJCC) tumor-node-metastasis (TNM) staging system (2). The TNM staging system continues to base the pathologic N category (pN) definitions on the absolute number of regional metastatic lymph nodes (3). However, the pN stage could have a stage migration because the number of metastatic lymph nodes can change according to the number of lymph nodes examined (eLNs). Meanwhile, the number of eLNs is affected by various factors, including the extent of lymphadenectomy, types of gastrectomy, characteristics of tumor, and skills of operators, thus many factors could influence the prognostic performance of the N staging system. Considering this, previous studies have proposed modifications to the TNM staging system by including the positive lymph node ratio (LNR). Node ratio (Nr) is defined as the number of metastatic lymph nodes divided by the number of eLNs (4). Several studies further confirmed that the tumor-node ratio-metastasis (TNrM) staging system was more appropriate than the AJCC 7th TNM staging system, and the main interpretation for that involved stage migration (5–11). Recently, the AJCC 8th TNM staging system has made several modifications to the 7th TNM staging system, including inclusion of pN3b into the staging system and modifications of existing staging subgroups (3). Subsequently, populations from both the West and the East have validated the AJCC 8th TNM staging system's superiority over the AJCC 7th TNM staging system for patients with adequate eLNs (12–15). However, the validity and superiority of the AJCC 8th TNM staging system compared with the TNrM staging system remain unknown. On the other hand, patients with adequate eLNs will have a slight risk of stage migration (16). Therefore, it should be questioned whether the TNrM staging system was superior than the AJCC 8th TNM staging system when predicting survival for patients with adequate eLNs.

In the light of these considerations, the aims of our present study were the following: (1) to modify the AJCC 8th TNM staging system based on the Nr; (2) to investigate whether the TNrM staging system was superior to the AJCC 8th TNM staging system to predict survival for GC patients regardless of the number of eLNs.

## Materials and Methods

### Patients

In this retrospective analysis, patients who underwent gastrectomy in the Surveillance, Epidemiology, and End Results (SEER) database with at least 18 years of age with histology-confirmed GC (ICD-O-3: M-8140/3, M-8142/3 to M-8145/3, M-8210/3, M-8211/3, M-8255/3, M-8260/3 to M-8263/3, M-8310/3, M-8323/3) were included. The detailed included process for GC patients is shown in Supplemental Figure 1. Finally, a total of 17,187 GC patients were included for this study. Further, these patients were classified into training set and validation set based on the SEER registries following our previous study (17). The median follow-up time for training and validation sets was 162 and 100 months, respectively.

### Statistical Methods

**Figure 1:**
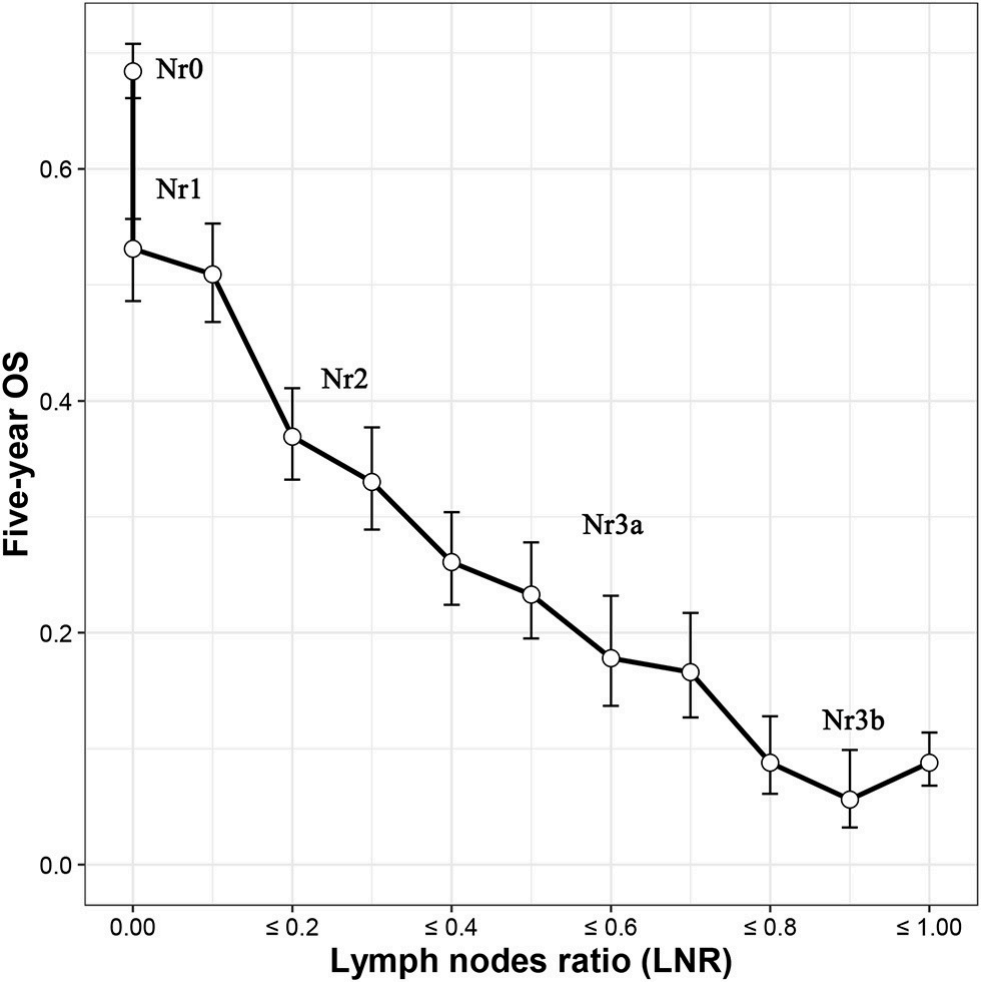
Five-year overall survival (OS) according to the lymph nodes ratio (LNR). Five Nr categories (Nr0–3b) were established by combining the neighborhood survival curves (Nr0: LNR = 0, eLNs ≤ 9; Nr1: LNR = 0, eLNs > 9, or 0 < LNR ≤ 0.1; Nr2: 0.1 < LNR ≤ 0.3; Nr3a: 0.3 < LNR ≤ 0.7; and Nr3b: LNR > 0.7). *eLNs*, lymph nodes examined.

To build the Nr staging system, we determined the cutoff values of the LNR as being where the Nr subgroups showed the 5-year overall survival (OS) that was the most similar. A unique stratification was developed for node-negative GC patients because of the fact that the LNR value for these patients are always the same, which will not be influenced by the number of eLNs. The optimal number of eLNs that determined the most significantly survival difference for node-negative GC patients was 9 (Supplemental Figure 2) using the X-tile software (http://www.tissuearray.org) (18). Then, 5 Nr stages were established by combining the neighborhood survival curves according to the 5-year OS (Nr0: LNR = 0, eLNs > 9; Nr1: LNR = 0, eLNs ≤ 9, or 0 < LNR ≤ 0.1; Nr2: 0.1 < LNR ≤ 0.3; Nr3a: 0.3 < LNR ≤ 0.7; and Nr3b: LNR > 0.7; Figure 1). In order not to further complicate an already complex staging system, the TNrM staging system was established by combining the same pT and pM from the AJCC 8th TNM staging system with the Nr instead of the pN (Supplemental Table 1).

The OS probabilities were calculated by the Kaplan-Meier method, and the different OS probabilities between groups were examined by log-rank test. The likelihood ratio test within a Cox regression model was used to assess the homogeneity of the staging system. Harrell's concordance index (C-index) was used to assess the relative discriminatory ability of the staging system, and a value closer to 1 means a higher predictive ability. Furthermore, the Akaike information criterion (AIC) within a Cox regression model was also used to measure the discriminatory ability of the staging system, and a smaller AIC value means a more reasonable model for prognosis (19). All statistical analyses were performed using R-3.4 software (http://www.r-project.org). *P* < 0.05 (two-sided) was considered statistically significant.

## Results

### Patient Demographics and Tumor Characteristics

The included process for GC patients is shown in Supplemental Figure 1. Finally, 17,187 GC patients with complete data for staging were identified. Of these, 7,660 patients were classified into the training set and 9,527 patients were classified into the validation set. The median number of eLNs for the training set was 12 (interquartile range [IQR],7–20), and for the validation set it was 13 (IQR, 7–20). The median OS time for training set was 38 (IQR,13–129) months and validation set was 42 (IQR, 13–149) months. Detailed characteristics of GC patients for the training and validation sets are summarized in Supplemental Table 2.

### Prognostic Performance of Different Staging Systems in the Training Set

For the training set, each pN category (pN0–3b) was stratified into Nr subgroups. Each pN category (pN0–3b) was found to contain subgroups of patients with significantly heterogeneous OS (all *P* < 0.001; Table 1). Similarly, each Nr category (Nr0–3b) was stratified into pN subgroups; only the Nr3b category was found to contain subgroups of patients with significantly heterogeneous OS (*P* < 0.001; Table 1). Moreover, the OS of each pN category (pN0–3b) for patients with > 15 eLNs was significantly better than that of patients with ≤15 eLNs (all *P* < 0.001; Table 2). Conversely, the significantly different OS was found in the Nr0, Nr2, and Nr3b categories for patients with >15 eLNs and patients with ≤15 eLNs (Nr0: *P* = 0.028; Nr2: *P* = 0.049; Nr3b: *P* = 0.008; Table 2), and the OS of Nr1and Nr3a categories for patients > 15 eLNs was similar to that of patients with ≤15 eLNs (all *P* > 0.05; Table 2). These results indicated that compared with the pN categories, Nr categories represent patients with more homogeneous OS. The Nr staging system had a higher likelihood ratio χ^2^ value and thus also represented better homogeneity (Table 3).

**Table 1:** Five-year overall survival by the AJCC 8th N staging system and the Nr staging system for the training set.

**8th pN stage**	**Nr stage**					
	**Nr0** **68.4% (1671)**	**Nr1** **52.5% (2179)**	**Nr2** **35.2% (1138)**	**Nr3a** **22.0% (1528)**	**Nr3b** **8.2% (1144)**	***P*-value**
pN0 60.7% (3240)^a^	68.4% (1671)	53.1% (1569)				<0.001
pN1 36.3% (1471)		51.3% (549)	33.8% (543)	21.2% (247)	15.9% (132)	<0.001
pN2 26.5% (1369)		45.2% (60)	37.6% (483)	22.5% (565)	11.7% (261)	<0.001
pN3a 17.4% (1170)		– (1)^b^	32.1% (110)	23.7% (589)	6.1% (470)	<0.001
pN3b 7.4% (410)			– (2)^b^	13.2% (127)	4.4% (281)	<0.001
*P*-value	–	0.538	0.052	0.383	<0.001	

*AJCC, American Joint Committee on Cancer*.

a *Five-year overall survival was calculated by Kaplan-Meier method, followed the number of patients*.

b *Five-year overall survival was not calculated for this subgroup because of limited number of patients*.

**Table 2:** Five-year OS by the AJCC 8th N staging system and the Nr staging system according to the number of eLNs.

**8th pN stage**	**No. of eLNs**	**Five-year OS**	***P*-value**	**Nr stage**	**No. of eLNs**	**Five-year OS**	***P*-value**
pN0 60.7% (3240)^a^	eLNs > 15	70.5% (1000)	reference	Nr0 68.4% (1671)	eLNs > 15	70.5% (1000)	reference
	eLNs ≤ 15	56.7% (2240)	< 0.001		eLNs ≤ 15	65.5% (671)	0.028
pN1 36.3% (1471)	eLNs > 15	53.7% (409)	reference	Nr1 52.5% (2179)	eLNs > 15	54.5% (420)	reference
	eLNs ≤ 15	30.0% (1062)	< 0.001		eLNs ≤ 15	52.1% (1759)	0.194
pN2 26.5% (1369)	eLNs > 15	38.8% (452)	reference	Nr2 35.2% (1138)	eLNs > 15	37.9% (505)	reference
	eLNs ≤ 15	20.9% (917)	< 0.001		eLNs ≤ 15	33.3% (633)	0.049
pN3a 17.4% (1170)	eLNs > 15	23.9% (623)	reference	Nr3a 22.0% (1528)	eLNs > 15	22.4% (610)	reference
	eLNs ≤ 15	10.2% (547)	< 0.001		eLNs ≤ 15	21.7% (918)	0.510
pN3b 7.4% (410)	eLNs > 15	7.4% (410)	–	Nr3b 8.2% (1144)	eLNs > 15	5.0% (359)	reference
					eLNs ≤ 15	9.6% (785)	0.008

*AJCC, American Joint Committee on Cancer; eLNs, lymph nodes examined; OS, overall survival*.

a *Five-year OS was calculated by Kaplan-Meier method, followed the number of patients*.

**Table 3:** Comparison of the performance of different staging systems.

**Staging system**	**Likelihood ratio χ^2^**	**C-index (95% CI)**	***P-*****value^a^**		**AIC**
**TRAINING SET**					
pN stage	1069.08	0.641 (0.633–0.649)	reference		92401.84
Nr stage	1514.53	0.666 (0.658–0.674)	< 0.001		91956.39
7th TNM stage	1447.85	0.672 (0.664–0.680)	reference		92027.07
8th TNM stage	1470.21	0.671 (0.663–0.679)	0.504	reference	92004.71
TNrM stage	1862.41	0.688 (0.680–0.696)	< 0.001	< 0.001	91612.51
**GROUP 1 (eLNs** **≤** **15)**					
pN stage	662.18	0.635 (0.625–0.645)	reference		57255.01
Nr stage	823.68	0.651 (0.641–0.661)	< 0.001		57095.51
7th TNM stage	979.68	0.673 (0.663–0.683)	reference		56238.48
8th TNM stage	976.83	0.673 (0.663–0.683)	0.610	reference	56946.37
TNrM stage	1106.88	0.682 (0.672–0.692)	< 0.001	< 0.001	56816.32
**GROUP 2 (eLNs** **>** **15)**					
pN stage	624.66	0.678 (0.664–0.692)	reference		27532.93
Nr stage	681.31	0.685 (0.671–0.799)	< 0.001		27476.28
7th TNM stage	638.71	0.690 (0.676–0.704)	reference		27522.88
8th TNM stage	713.25	0.694 (0.680–0.708)	0.079	reference	27448.34
TNrM stage	760.97	0.700 (0.686–0.714)	< 0.001	< 0.001	27400.62
**VALIDATION SET**					
pN stage	1325.43	0.646 (0.638–0.654)	reference		98283.28
Nr stage	1892.79	0.674 (0.666–0.682)	< 0.001		97715.92
7th TNM stage	1820.09	0.677 (0.669–0.685)	reference		97792.62
8th TNM stage	1821.90	0.676 (0.668–0.684)	0.504	reference	97790.81
TNrM stage	2334.05	0.696 (0.688–0.704)	< 0.001	< 0.001	97278.66
**GROUP 1 (eLNs** **≤** **15)**					
pN stage	844.05	0.640 (0.630–0.650)	reference		60901.31
Nr stage	1024.0	0.656 (0.646–0.666)	< 0.001		60723.29
7th TNM stage	1225.28	0.679 (0.669–0.689)	reference		60526.08
8th TNM stage	1231.46	0.678 (0.668–0.688)	0.205	reference	60519.91
TNrM stage	1370.3	0.687 (0.677–0.697)	< 0.001	< 0.001	60380.99
**GROUP 2 (eLNs** **>** **15)**					
pN stage	784.81	0.693 (0.679–0.707)	reference		29499.20
Nr stage	867.43	0.700 (0.686–0.714)	< 0.001		29416.58
7th TNM stage	854.00	0.700 (0.686–0.714)	reference		29434.01
8th TNM stage	916.25	0.708 (0.694–0.722)	< 0.001	reference	29371.76
TNrM stage	989.72	0.714 (0.700–0.728)	< 0.001	0.002	29298.29

*AJCC, American Joint Committee on Cancer; TNM, tumor-node-metastasis; TNrM, tumor-node ratio-metastasis; CI, confidence interval; AIC, Akaike information criterion; eLNs, lymph nodes examined*.

a P-value was calculated by R package “compareC.”

**Figure 2:**
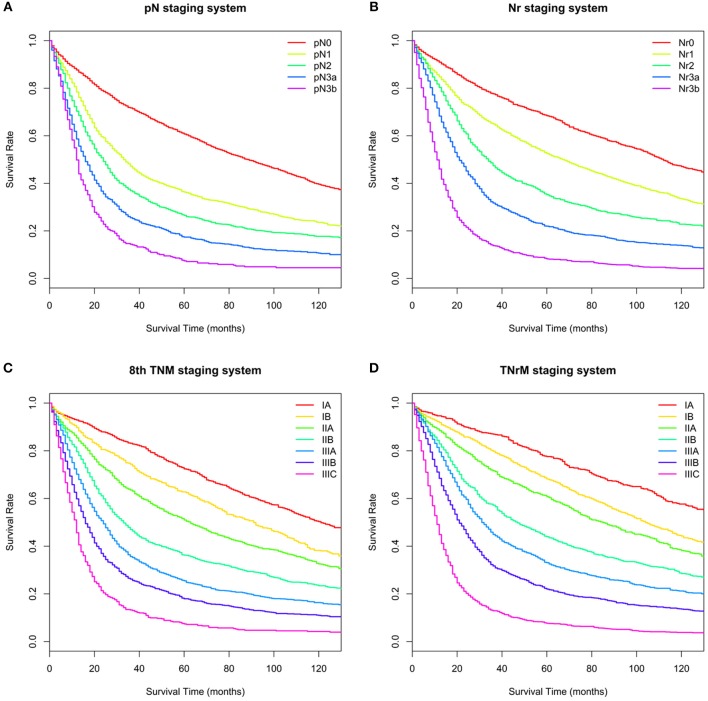
Overall survival (OS) for the training set based on different staging systems. **(A)** pN staging system. Five-year OS for the pN categories (pN0–3b) was 60.7, 36.3, 26.5, 17.4, and 7.4%, respectively. **(B)** Nr staging system. Five-year OS for the Nr categories (Nr0–3b) was 68.4, 52.5, 35.3, 22.0, and 8.2%, respectively. **(C)** 8th TNM staging system. Five-year OS for the 8th TNM stages (IA–IIIC) was 72.4, 62.8, 51.0, 36.3, 25.5, 18.0, and 7.4%, respectively. **(D)** TNrM staging system. Five-year OS for the TNrM stages (IA–IIIC) was 77.6, 68.2, 60.7, 44.0, 33.1, 22.0, and 7.7%, respectively.

The survival curves for the training set are shown in Figure 2. The C-index value of the Nr staging system was significantly higher than that of the pN staging system to predict survival for GC patients (C-index: 0.666 vs. 0.641, *P* < 0.001; Table 3). The TNrM staging system was constructed on the basis of the same pT and pM with the new Nr (Supplemental Table 1). Furthermore, we compared the prognostic performance for these two staging systems. The TNrM staging system performed better than the AJCC 8th TNM staging system to predict survival for GC patients (C-index: 0.688 vs. 0.671, *P* < 0.001; Table 3). In order to evaluate the influence of the number of eLNs on the prognostic performance of these two staging systems, we classified the GC patients into 2 groups according to the number of eLNs: group 1, eLNs ≤ 15; group 2, eLNs > 15. The cutoff point of 15 was chosen because of the fact that 15 lymph nodes was the landmark of eLNs recommended by the National Comprehensive Cancer Network (NCCN) to avoid stage migration (20). For GC patients with ≤ 15 eLNs, the Nr staging system and the TNrM staging system performed better than the AJCC staging system to predict survival for these GC patients (C-index: Nr vs. pN, 0.651 vs. 0.635, *P* < 0.001; TNrM vs. TNM: 0.682 vs. 0.673, *P* < 0.001; Table 3). Similarly, for GC patients with >15 eLNs, the Nr staging system and the TNrM staging system represented patients with better prognostic identification than the AJCC staging system (C-index: Nr vs. pN: 0.685 vs. 0.678, *P* < 0.001; TNrM vs. TNM: 0.700 vs. 0.694, *P* < 0.001; Table 3).

### Prognostic Performance of Different Staging Systems in the Validation Set

**Figure 3:**
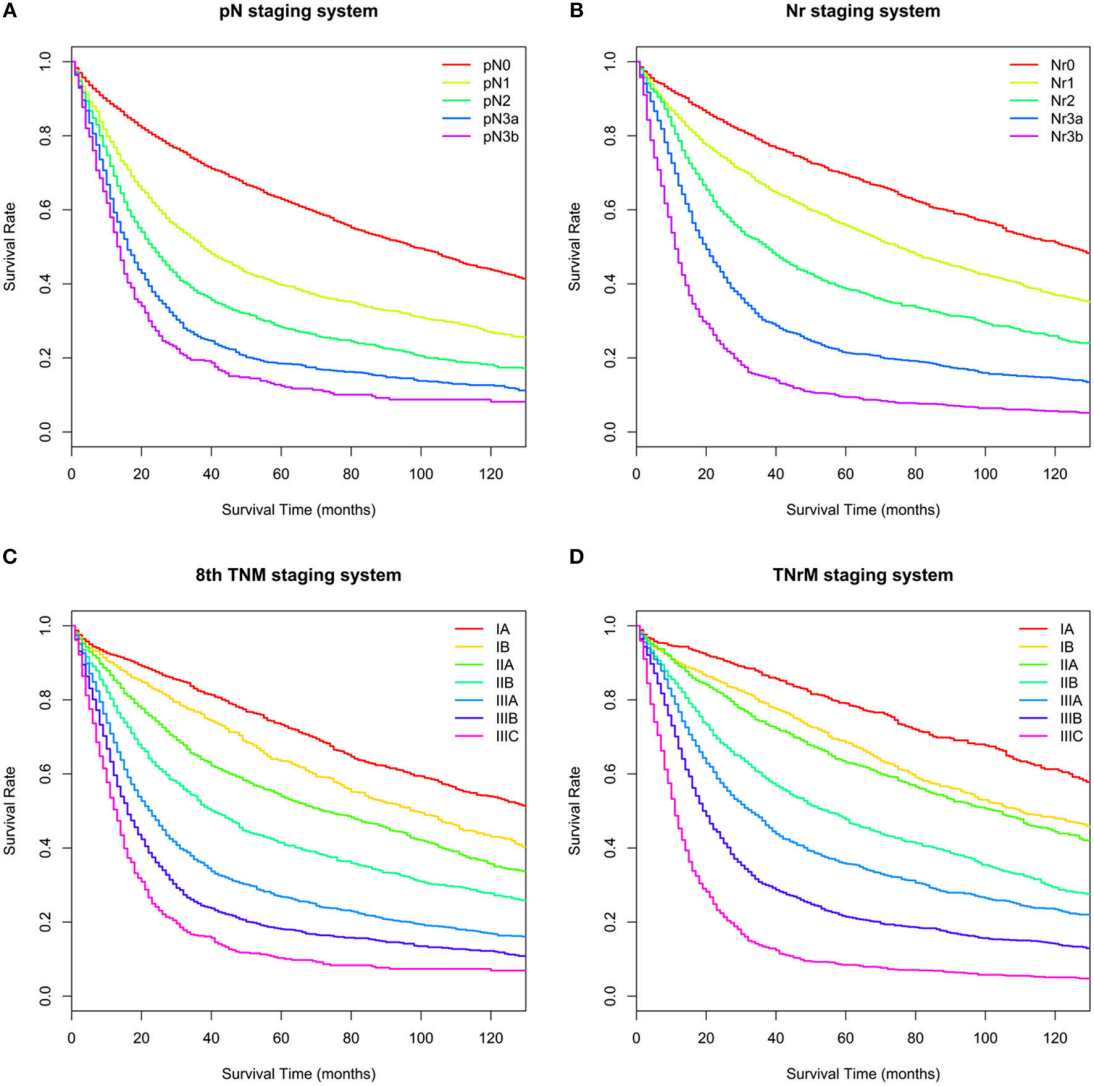
Overall survival (OS) for the validation set based on different staging systems. **(A)** pN staging system. Five-year OS for the pN categories (pN0–3b) was 62.9, 40.0, 28.3, 18.5, and 12.5%, respectively. **(B)** Nr staging system. Five-year OS for the Nr categories (Nr0–3b) was 69.4, 55.8, 38.7, 21.4, and 9.4 %, respectively. **(C)** 8th TNM staging system. Five-year OS for the 8th TNM stages (IA–IIIC) was 73.3, 63.5, 53.9, 41.4, 26.8, 18.1, and 10.3%, respectively. **(D)** TNrM staging system. Five-year OS for the TNrM stages (IA–IIIC) was 79.0, 68.5, 63.1, 47.7, 35.8, 21.4, and 8.5, respectively.

To validate results from the training set, we conducted similar analyses in the validation set. The survival curves for the validation set are shown in Figure 3. The C-index values of the Nr and TNrM staging systems were significantly higher than that of the AJCC 8th TNM staging system to predict survival for GC patients (C-index: Nr vs. pN: 0.674 vs. 0.646, *P* < 0.001; TNrM vs. TNM: 0.696 vs. 0.676, *P* < 0.001; Table 3). Similarly, the C-index values of the Nr and TNrM staging systems were significantly higher than that of the 8th TNM staging system to predict survival for GC patients with ≤15 eLNs (C-index: Nr vs. pN: 0.656 vs. 0.640, *P* < 0.001; TNrM vs. TNM: 0.687 vs. 0.678, *P* < 0.001; Table 3), as well as for GC patients with >15 eLNs (C-index: Nr vs. pN: 0.700 vs. 0.693, *P* < 0.001; TNrM vs. TNM: 0.714 vs. 0.708, *P* = 0.002; Table 3).

## Discussion

An accurate staging system is important for prognostic assessment and tailoring a treatment plan for GC patients. Several studies have validated that the main superiority of 8th TNM staging system compared with the 7th TNM staging system was to include pN3b into the staging system (12–15). In our study, patients with pN3a had a significantly better OS than those with pN3b, and the 8th TNM staging system was superior to the 7th TNM staging system when predicting survival for GC patients with >15 eLNs, these results were consistent with previous studies (12–15). However, for all patients (included those with ≤ 15 eLNs), the AJCC 8th TNM staging system was comparable with the 7th TNM staging system, which suggested that the number of eLNs had an influence on the capacity of AJCC TNM prognosis assessment.

Previous studies suggested that the TNrM staging system may better stratify patients' survival than the AJCC TNM staging system (5–11), which was validated in our study. The TNrM staging system satisfied three ideal models of lymph node staging conditions: (1) similar survival rates within a stage group, (2) decreased survival rates with increasing stages, and (3) difference in survival between different stages (21). The main superiority of the TNrM staging system compared with the AJCC TNM staging system is to minimize the stage migration phenomenon. The node-negative patients with fewer than 10 eLNs may not truly be node-negative but rather understaged (17, 22). The stage pN1 can be upstaged to pN2 or even pN3 as more lymph nodes are examined. Furthermore, it is impossible to be categorized as pN3b if fewer than 16 lymph nodes are examined. On the contrary, the TNrM staging system has been proposed to reduce the difference of OS within subgroups with ≤ 15 and >15 eLNs (5, 11). Similar results were found in our study: the OS of each N category (pN0–3a) patients with >15 eLNs was significantly better than that of patients with ≤15 eLNs. Conversely, significantly different OS was only found in the Nr0 and Nr3b categories for patients with >15 eLNs and ≤15 eLNs.

However, the risk of stage migration will decrease as the number of eLNs increased. It should be questioned whether the TNrM staging system performed better than the AJCC 8th TNM staging system for patients with adequate eLNs. It is clear that if the TNM staging system performed as well as the TNrM staging system, the application value of the TNrM staging system would be limited as most patients in Asian had >15 eLNs (3, 23) and the rate of patients with >15 eLNs was increasing in Western (24) countries. Therefore, we conducted a population-based study to investigate whether the TNrM staging system maintained its prognostic power for patients with adequate eLNs. For patients with adequate eLNs, the TNrM staging system still had a better prognostic performance than the AJCC TNM staging system in terms of homogeneity and discriminatory ability. Besides the mechanism stage migration, these results may be related to the fact that the number of lymph nodes itself correlates with survival. A randomized controlled trial suggested that an increase in the number of lymph nodes did not correlate with a change in the percent of node-positive patients or the mean number of metastatic lymph nodes (25). Parsons et al. further confirmed that patients with a high number of eLNs were only slightly more likely to be node-positive, while these patients experienced significantly better OS compared with those with fewer eLNs (16). Several studies have hypothesized that the number of lymph nodes might reflect the tumor-host relationship, and a higher number of lymph nodes dissected may simply reflect a host lymphocytic reaction to the tumor, which is associated with long survival (26–28). However, it is impossible for us to answer where the risk of being understaged ends and where a true benefit of number of eLNs begins.

To the best of our knowledge, this was the first study to directly modify the AJCC 8th TNM staging system based on the Nr when predicting survival for GC patients using a large database with long-term follow-up. Compared with previous research (5–11), there were two main modifications in this study. Firstly, node-negative GC patients were classified into Nr0 and Nr1 based on the number of eLNs. Further, we proved the validity of this classification. Secondly, the TNrM staging system could be improved with modifications of staging subgroups. However, in order not to further complicate an already complex staging model, the TNrM staging system was established by combining the same pT and pM with the Nr instead of the pN. Still, several limitations should be noted in this study. First, all patients were selected from the SEER database, which may restrict it to other populations. The number of eLNs for gastric cancer patients in Asia was more than that in Western countries. However, our study verified that the TNrM staging system performed better than the TNM staging system even for patients with adequate eLNs. Thus, we believe similar results will be found in Asian populations. On the other hand, these cutoff points may not be optimal for the other cohorts of patients, and modification of the LNR intervals may be needed. Secondly, patients without negative lymph nodes may have a risk of upstaged because the Nr stage will be the same (Nr3b), which will not be influenced by the number of eLNs. However, we believed patients without negative lymph nodes have a higher risk testing positive for residual lymph nodes, which was associated with worse survival rates. These patients were staged as Nr3b, and this may accurately reflect the risk of residual positive lymph nodes. Thirdly, unlike neoadjuvant radiotherapy, we were unable to determine whether the patients received neoadjuvant chemotherapy that might affect pathological judgment. Given that the standard of neoadjuvant has been concurrent chemoradiotherapy, it is reasonable to assume that patients received combined neoadjuvant chemoradiotherapy. We believe that few patients who received only chemotherapy were included in our study. Despite these limitations, the present study was significant because the TNrM staging system resulted in more GC patients being accurately and discriminatively staged without overcomplicating the TNM staging system.

## Conclusions

The TNrM staging system predicted survival more accurately and discriminatively than the AJCC 8th TNM staging system for GC patients regardless of the number of eLNs, which should be taken into account as a supplemental staging system when predicting survival for GC patients, especially for those with limited eLNs. However, this result should be validated in other populations.

## Data Availability

Publicly available datasets were analyzed in this study. This data can be found here: https://seer.cancer.gov/.

## Ethics Statement

All patients in this study were collected from the SEER database, and we received permission for using the data.

## Author Contributions

Z-LY, J-HL, and C-XW designed this study. Z-LY, M-HZ, X-JH, and Q-WL performed the search and collected data. C-XW rechecked data. Z-LY and J-HL performed analysis. Z-LY wrote the manuscript. All authors reviewed the manuscript.

### Conflict of Interest Statement

The authors declare that the research was conducted in the absence of any commercial or financial relationships that could be construed as a potential conflict of interest.
