# Supplementary material for: Modified American Joint Committee on Cancer Tumor-Node-Metastasis Staging System Based on the Node Ratio Can Further Improve the Capacity of Prognosis Assessment for Gastric Cancer Patients
Source: Front Oncol. 2019 May 3;9:329. doi: 10.3389/fonc.2019.00329 (PMC6509971; doi:10.3389/fonc.2019.00329)
Supplement: Supplementary file 1 [file Table_1.DOCX]

**Modified American Joint Committee on Cancer tumor-node-metastasis staging system based on the node ratio can further improve the capacity of prognosis assessment for gastric cancer patients**

**Short running head:** **Staging for gastric cancer**

**Supplemental Figures:** 2 Figures.

**Supplemental Tables:** 2 Tables.


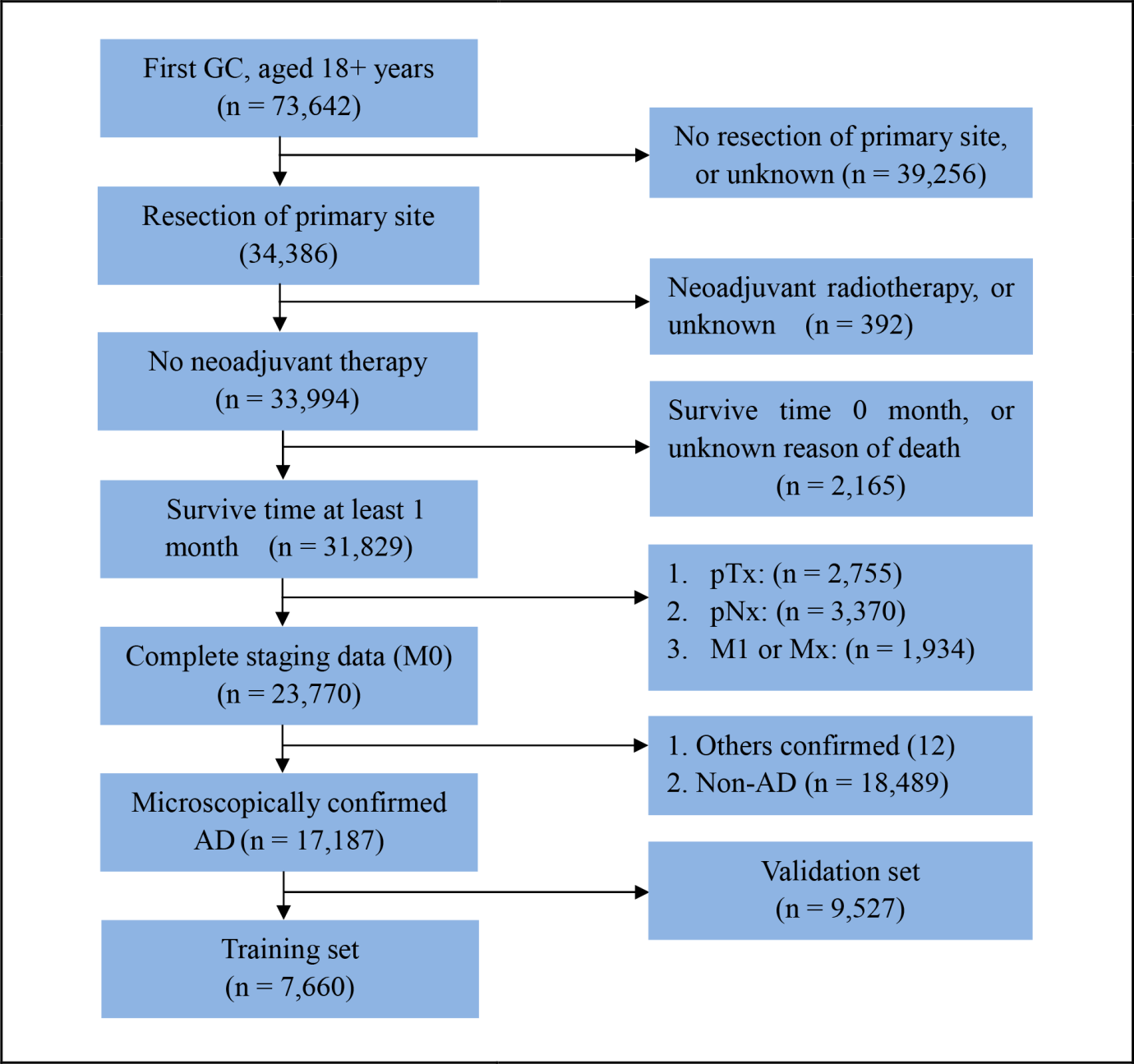


**Supplemental Figure 1** Flowchart of gastric cancer (GC) patients included process. *AD*, adenocarcinoma.


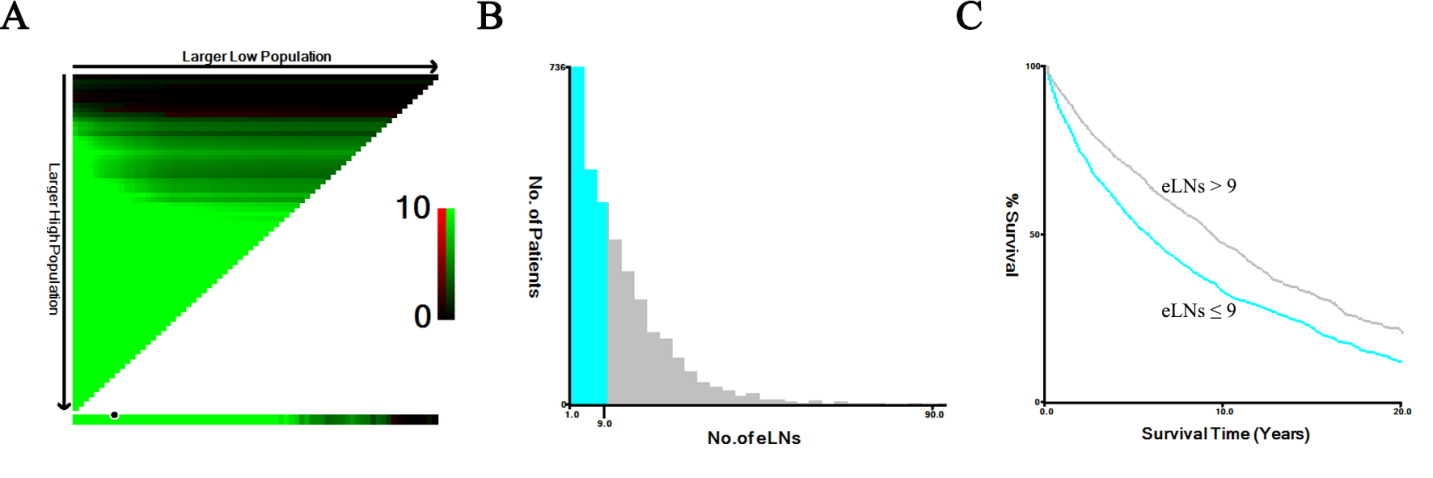
**Supplemental Figure 2** The cutoff point for node-negative gastric cancer patients of training set. **A** X-tile plot. **B** Histogram plot. **C** Kaplan-Meier plot. The most discriminative cutoff number of lymph nodes examined (eLNs) for node-negative gastric cancer patients was 9 (*P* < 0.001). *eLNs*, lymph nodes examined.

**Supplemental Table 1** The AJCC TNM staging system and the TNrM staging system

| 8th TNM stage | N0 | N1 | N2 | N3a | N3b |
| --- | --- | --- | --- | --- | --- |
| T1 | IA | IB | IIA | IIB | IIIB |
| T2 | IB | IIA | IIB | IIIA | IIIB |
| T3 | IIA | IIB | IIIA | IIIB | IIIC |
| T4a | IIB | IIIA | IIIA | IIIB | IIIC |
| T4b | IIIA | IIIB | IIIB | IIIC | IIIC |
| TNrM stage | Nr0 | Nr1 | Nr2 | Nr3a | Nr3b |
| T1 | IA | IB | IIA | IIB | IIIB |
| T2 | IB | IIA | IIB | IIIA | IIIB |
| T3 | IIA | IIB | IIIA | IIIB | IIIC |
| T4a | IIB | IIIA | IIIA | IIIB | IIIC |
| T4b | IIIA | IIIB | IIIB | IIIC | IIIC |

*AJCC*, American Joint Committee on Cancer; *TNM*, tumor-node-metastasis; *TNrM*, tumor-node ratio-metastasis.

**Supplemental Table 2** The clinicopathological features for gastric cancer patients

| Characteristic | All Patients | | Training set | | Validation set | |
| --- | --- | --- | --- | --- | --- | --- |
|  | No. of patients | Percentage  (%) | No. of patients | Percentage  (%) | No. of patients | Percentage  (%) |
| Age (years) |  |  |  |  |  |  |
| ≤ 60 | 4116 | 23.95 | 1732 | 22.61 | 2384 | 25.02 |
| ≤ 70 | 4422 | 25.73 | 1958 | 25.56 | 2464 | 25.86 |
| ≤ 80 | 5523 | 32.13 | 2497 | 32.60 | 3026 | 31.76 |
| > 80 | 3126 | 18.19 | 1473 | 19.23 | 1653 | 17.35 |
| Sex |  |  |  |  |  |  |
| Male | 10122 | 58.89 | 4488 | 58.59 | 5634 | 59.14 |
| Female | 7065 | 41.11 | 3172 | 41.41 | 3893 | 40.86 |
| Ethnicity |  |  |  |  |  |  |
| White | 9785 | 56.93 | 4214 | 55.01 | 5571 | 58.48 |
| Black | 2678 | 15.58 | 1172 | 15.30 | 1506 | 15.81 |
| Asian or Pacific Islander | 4524 | 26.32 | 2194 | 28.64 | 2330 | 24.46 |
| Others | 200 | 1.16 | 80 | 1.04 | 120 | 1.26 |
| Location |  |  |  |  |  |  |
| Fundus of stomach | 724 | 4.21 | 333 | 4.35 | 391 | 4.10 |
| Body of stomach | 1854 | 10.79 | 743 | 9.70 | 1111 | 11.66 |
| Gastric antrum | 6147 | 35.77 | 2678 | 34.96 | 3469 | 36.41 |
| Pylorus | 1026 | 5.97 | 414 | 5.40 | 612 | 6.42 |
| Lesser curvature of stomach, NOS | 2980 | 17.34 | 1373 | 17.92 | 1607 | 16.87 |
| Greater curvature of stomach, NOS | 1132 | 6.59 | 542 | 7.08 | 590 | 6.19 |
| Overlapping lesion of stomach | 1618 | 9.41 | 771 | 10.07 | 847 | 8.89 |
| Stomach, NOS | 1706 | 9.93 | 806 | 10.52 | 900 | 9.45 |
| Grade |  |  |  |  |  |  |
| Well/moderately differentiated | 6233 | 36.27 | 2804 | 36.61 | 3429 | 35.99 |
| Poorly/undifferentiated | 10348 | 60.21 | 4503 | 58.79 | 5845 | 61.35 |
| Unknown | 606 | 3.53 | 353 | 4.61 | 253 | 2.66 |
| Tumor size |  |  |  |  |  |  |
| ≤ 2 cm | 2662 | 15.49 | 1097 | 14.32 | 1565 | 16.43 |
| ≤ 5 cm | 6496 | 37.80 | 2835 | 37.01 | 3661 | 38.43 |
| > 5 cm | 5794 | 33.71 | 2621 | 34.22 | 3173 | 33.31 |
| Unknown | 2235 | 13.00 | 1107 | 14.45 | 1128 | 11.84 |
| AJCC 8th pT |  |  |  |  |  |  |
| T1 | 4017 | 23.37 | 1746 | 22.79 | 2271 | 23.84 |
| T2 | 2217 | 12.90 | 922 | 12.04 | 1295 | 13.59 |
| T3 | 6227 | 36.23 | 2858 | 37.31 | 3369 | 35.36 |
| T4a | 3201 | 18.62 | 1372 | 17.91 | 1829 | 19.20 |
| T4b | 1525 | 8.87 | 762 | 9.95 | 763 | 8.01 |
| AJCC 8th pN |  |  |  |  |  |  |
| pN0 | 7402 | 43.07 | 3240 | 42.30 | 4162 | 43.69 |
| pN1 | 3212 | 18.69 | 1471 | 19.20 | 1741 | 18.27 |
| pN2 | 3091 | 17.98 | 1369 | 17.87 | 1722 | 18.07 |
| pN3a | 2569 | 14.95 | 1170 | 15.27 | 1399 | 14.68 |
| pN3b | 913 | 5.31 | 410 | 5.35 | 503 | 5.28 |
| Node ratio (Nr) |  |  |  |  |  |  |
| Nr0 | 3900 | 22.69 | 1671 | 21.81 | 2229 | 23.40 |
| Nr1 | 4921 | 28.63 | 2179 | 28.45 | 2742 | 28.78 |
| Nr2 | 2609 | 15.18 | 1138 | 14.86 | 1471 | 15.44 |
| Nr3a | 3340 | 19.43 | 1528 | 19.95 | 1812 | 19.02 |
| Nr3b | 2417 | 14.06 | 1144 | 14.93 | 1273 | 13.36 |
| Surgery |  |  |  |  |  |  |
| Gastrectomy, NOS | 13925 | 81.02 | 6198 | 80.91 | 7727 | 81.11 |
| Near total/total | 3262 | 18.98 | 1462 | 19.09 | 1800 | 18.89 |
| Lymph nodes examined |  |  |  |  |  |  |
| ≤ 15 | 10537 | 61.31 | 4766 | 62.22 | 5771 | 60.58 |
| > 15 | 6650 | 38.69 | 2894 | 37.78 | 3756 | 39.42 |
| Adjuvant therapy |  |  |  |  |  |  |
| No | 10812 | 62.91 | 5095 | 66.51 | 5717 | 60.01 |
| Chemotherapy | 2368 | 13.78 | 929 | 12.13 | 1439 | 15.10 |
| Radiotherapy | 427 | 2.48 | 190 | 2.48 | 237 | 2.49 |
| Chemoradiotherapy | 3580 | 20.83 | 1446 | 18.88 | 2134 | 22.40 |

*AJCC*, American Joint Committee on Cancer; *eLNs*, lymph nodes examined, *NOS*, not otherwise specified.
